# Photogating Effect-Driven Photodetectors and Their Emerging Applications

**DOI:** 10.3390/nano13050882

**Published:** 2023-02-26

**Authors:** Jihyun Shin, Hocheon Yoo

**Affiliations:** Department of Electronic Engineering, Gachon University, Seongnam 13120, Republic of Korea

**Keywords:** phototransistors, heterostructure, charge trapping, neuromorphic devices, photogating

## Abstract

Rather than generating a photocurrent through photo-excited carriers by the photoelectric effect, the photogating effect enables us to detect sub-bandgap rays. The photogating effect is caused by trapped photo-induced charges that modulate the potential energy of the semiconductor/dielectric interface, where these trapped charges contribute an additional electrical gating-field, resulting in a shift in the threshold voltage. This approach clearly separates the drain current in dark versus bright exposures. In this review, we discuss the photogating effect-driven photodetectors with respect to emerging optoelectrical materials, device structures, and mechanisms. Representative examples that reported the photogating effect-based sub-bandgap photodetection are revisited. Furthermore, emerging applications using these photogating effects are highlighted. The potential and challenging aspects of next-generation photodetector devices are presented with an emphasis on the photogating effect.

## 1. Introduction

As commonly used photodetectors, semiconductor-based photodetectors absorb photons originated by light irradiation and convert them into an electric current [1,2,3]. The ability of the photodetector is determined by how efficiently the device absorbs the target light and how sensitively and accurately it detects it [4]. One of the representative parameters for evaluating the performance of a photodetector is responsivity (*R*). Responsivity shows how sensitive the device is according to the intensity of the irradiated light. It can also be represented by an electric current converted from the light signal. Other photodetector parameters are described in Table 1 [5].

These electronic device-type photodetectors can be classified into photoconductors [6,7,8,9,10], photodiodes [11,12,13,14,15], and phototransistors [16,17,18,19,20,21,22,23,24], depending on their structure and operation principles. While photoconductors and photodiodes generate photocurrents in a two-terminal structure, phototransistors are based on a thin-film transistor (TFT) structure with three terminals having an additional electrode: a gate electrode, in which the electric fields can be modulated by an applied gate voltage (V_G_) bias, forming channel conductance [25,26,27,28,29]. Based on this principle, phototransistors can detect light and amplify the detected light signal without external devices [30,31,32]. For this reason, phototransistors have attracted considerable attention and have been developed using various active materials as a charge transport layer, such as metal oxides [33,34,35,36,37,38,39,40], polymers [41,42,43,44,45,46], small molecules [47,48,49,50,51,52,53,54,55,56], 2D materials [57,58,59,60,61,62,63,64], and carbon nanotubes [65,66,67,68].

The photoelectric effect is the general driving mechanism for operating phototransistors. Photo-excited carriers are generated when incident photons are absorbed in a material [69,70,71,72]. In this principle, the excited electrons and holes are dissociated from the valence band to the conduction band and vice versa with energy hν larger than the energy bandgap (E_g_) between the bands [73]. Thus, only light exposure with an energy hν larger than E_g_ can motivate the photodetection operation in these photoelectric effect-based devices.

Another mechanism for phototransistors to detect sub-bandgap rays is called the photogating effect. The mechanism of the photogating effect is explained by the ability of light to change the electronic structure of a material. When a material is exposed to light, photons can interact with electrons in the material to generate excited electrons and holes. These excited electrons and holes can diffuse into the material and change its electrical properties. Rather than photocurrent generation through photo-excited carriers, the photogating effect results from photoinduced trapped charges [25,74,75]. For example, the photogating effect is often related to the presence of impurities, known as trap states, within the material. These trap states can trap excited electrons and holes generated by light and alter the potential energy of the semiconductor/dielectric interface. These changes in charge distribution can contribute an additional electric gating field, leading changes in charge transport behavior, which is the shift in the threshold voltage (V_Th_). These trapped charges clearly distinguish the drain current under dark and light exposure [76,77,78,79]. As the photogating effect is obtained by generation due to the energy states inside the energy bandgap, the photogating effect can be used to enhance the photoresponse, even for sub-bandgap wavelengths.

Recent studies about the photogating effect have been reviewed according to different channel materials, but most of them have been focused on low dimensional materials. This paper reports recent efforts in photogating effect-based photodetectors involving various active materials, including perovskite, organics, compounds, polymers, and oxides, as well as 2D materials. The structure, material, and mechanism ideas driving the photogating effect to enhance the photodetection performances are summarized and classified by charge transport materials. In addition, studies that implemented the photogating effect in neuromorphic devices and optoelectronic memory are introduced, highlighting the potential of the photogating effect to be utilized more actively in next-generation applications beyond von Neumann without material limitation.

## 2. Recent Advances Using the Photogating Effect

### 2.1. Graphene-Based Photodetectors

**Figure 1:**
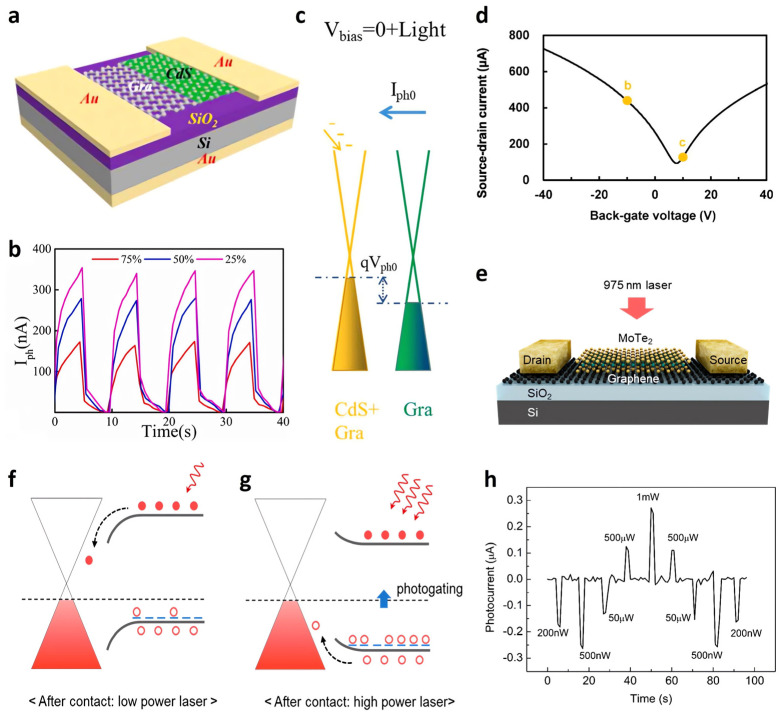
Graphene-based photodetector. (**a**) Graphene–CdS heterostructure schematic. (**b**) The I_Ph_ changes as the area of CdS covering graphene increased from 25% to 75%. (**c**) Schematic band diagram of a graphene–CdS heterostructure under light irradiation in V_bias_ = 0. (**d**) V_G_–I_D_ curve of a graphene-based photodetector using an InSb substrate under dark conditions. The lowest I_D_ was obtained at a V_G_ of approximately 7.7 V. (**e**) Schematic diagram of a graphene–MoTe_2_ heterostructure. Schematic band diagram of a graphene–MoTe_2_ heterostructure at (**f**) a low intensity of light and (**g**) a high intensity of light. (**h**) I_Ph_ (I_Laser_–I_Dark_) at various laser powers as a function of time. (**a**–**c**) Reproduced with permission from [80]. Copyright Elsevier, 2022. (**d**) Reproduced with permission from [83]. Copyright AIP Publishing, 2018. (**e**–**h**) Reproduced with permission from [84]. Copyright American Chemical Society, 2020.

Graphene is used widely as an active channel material to induce photogating effects in photodetectors. In 2022, Gao et al. operated a graphene-based photogating effect detector at zero bias, reporting a responsivity (*R*) of 0.26 A·W^−1^ in visible light [80]. Adapting the asymmetric structure of a graphene channel using cadmium sulfide (CdS) film made this feasible (Figure 1a). Graphene covered with CdS nanocrystals acted as a *p*-doped channel. With light illumination, the photogenerated carriers were separated in the CdS nanocrystals. While the holes were trapped in the CdS, the electrons were injected into the graphene, and the Fermi level (E_F_) of the graphene increased, causing a photocurrent (I_Ph_) to flow (Figure 1c). Interestingly, as the area of CdS-covering graphene increased, the I_Ph_ decreased (Figure 1b). In general, Si is used widely as a substrate. On the other hand, this has limited the detection wavelengths of optoelectronics from the visible to near-infrared (NIR) because of the cut-off wavelength of Si [81,82]. In 2018, Fukushima et al. produced graphene-based photodetectors that detect middle-wavelength infrared (MWIR, 3–5 μm) spectral bands used in industry, military, and aerospace by substituting a Si substrate with indium antimonide (InSb) as the active layer [83]. Tetraethyl orthosilicate (TEOS)-SiO_2_ was adopted as a dielectric layer. Figure 1d shows that the source-drain current (I_D_) had the lowest value at approximately V_G_ = 7.7 V under dark conditions. This voltage corresponds to the Dirac point of graphene, indicating the photogating effect by the InSb active layer. When MWIR light was irradiated, photogenerated electrons of InSb were trapped at the trap sites of the TEOS–SiO_2_/InSb interface, acting as additional negative V_G_ and modulating the surface charge density of graphene. As a result, an ultrahigh *R* of 33.8 A·W^−1^ was achieved by irradiating with 4.6 μm MWIR light at 50 K. Kim et al. conducted another study on graphene-based optoelectronics in 2020. They reported a gradual and reversible transition between negative photoconductivity (NPC) and positive photoconductivity by a photogating effect in a single device [84]. NPC, whose photoconductivity is reduced by light, has low power consumption and a rapid frequency response. They implemented molybdenum ditelluride (MoTe_2_) and a graphene heterostructure as the active and channel layers, respectively (Figure 1e). Under 975 nm light irradiation, photo-excited hole carriers trapped in MoTe_2_ modulated the Fermi level of the MoTe_2_–graphene junction, lowering the graphene conductivity from the initial state to the NPC (Figure 1f). Interestingly, when the laser power was increased to more than 500 μW, the NPC was converted to positive photoconductivity due to changes in the graphene/MoTe_2_ Schottky junction with reverse-bending and hole carrier injection (Figure 1g,h). The transition from NPC to the positive photoconductivity and vice versa was indestructible.

### 2.2. TMD- and BP-Based Photodetectors

**Figure 2:**
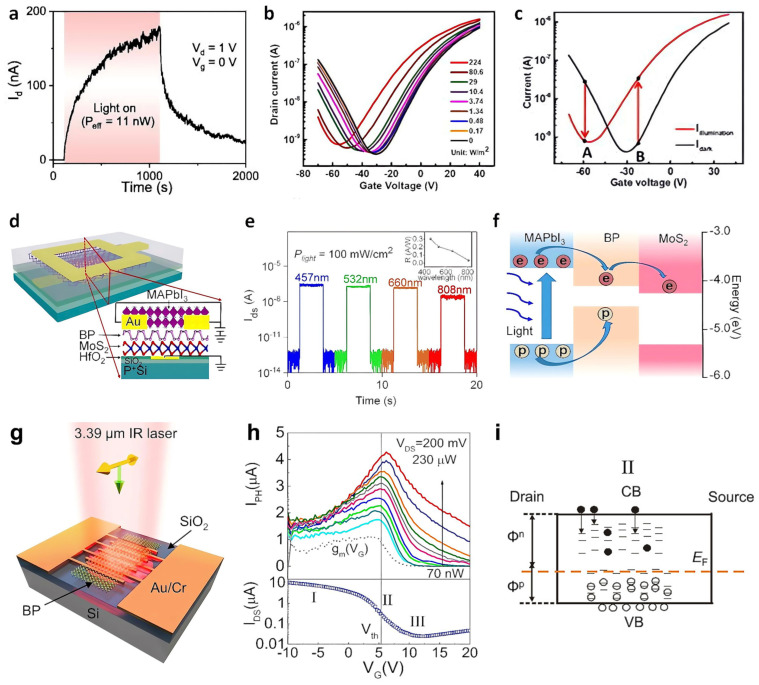
TMD-based photodetector. (**a**) Time evolution of the I_D_ under white LED light illumination (11 nW) when surface treated with O_3_ to form a WO_x_ (x < 3) layer on WSe_2_. (**b**) Transfer curve of the PtSe_2_ photodetector according to the light intensity with a 405 nm laser irradiation. (**c**) Transfer curve of the PtSe_2_ photodetector with (red curve) and without (dark curve) light irradiation. (**d**) Schematic diagram of the perovskite/BP/MoS_2_ photodiode. (**e**) Time evolution of the I_D_ (V_D_ = 0) with different wavelengths of 457, 532, 660, and 808 nm. The inset shows the corresponding *R*. (**f**) Schematic band diagram and photocarrier transfer of the device under laser illumination. (**g**) Scheme of the BP photodetector. (**h**) Upper panel: gate-dependent I_Ph_ measured according to the various intensities of 532 nm light. Lower panel: transfer curve corresponding to the upper panel for reference at V_D_ = 200 mV. (**i**) Schematics of the energy band for the state II (V_G_ ≈ V_Th_) of (**h**). The down arrows denote the carrier trapping process. The black dots and open circles represent electrons and holes, respectively. (**a**) Reproduced with permission from [91]. Copyright AIP Publishing, 2018. (**b**,**c**) Reproduced with permission from [94]. Copyright AIP Publishing, 2021. (**d**–**f**) Reproduced with permission from [88]. Copyright American Chemical Society, 2019. (**g**–**i**) Reproduced with permission from [103]. Copyright American Chemical Society, 2016.

Two-dimensional transition metal dichalcogenides (2D TMDs) have the advantages of a tunable bandgap, a layer number-dependent band structure, easy fabrication, ultrastability, a high on-current (I_On_)/off-current (I_Off_) ratio, high electron mobility (μ), and high *R* [4,85,86,87]. On the other hand, their slow response requires charge-trapping layers, such as adsorbates and oxides [5,88,89,90]. Integrating an oxide layer in TMDs is not easy, but surface oxidation of atomically thin TMDs was one of the breakthroughs. In 2018, Yamamoto et al. treated a tungsten diselenide (WSe_2_) surface with O_3_ to form a self-limiting oxide layer, which is WO_x_ [91]. It served as a photogating medium, providing electron trap sites and extending the carrier lifetime. As a result, the I_Ph_ showed persistent photoconductivity (PPC) behavior (Figure 2a). A high *R* of 3663 A·W^−1^ was obtained using white light with an intensity of 1.1 nW. Group-10 TMDs have excellent optical and electronic properties, which respond to light in a broadband wavelength range from visible to mid-infrared, and are appropriate for ultrathin and flexible photodetectors [92,93]. In 2021, Yang et al. produced Se vacancies as hole trap sites by tape exfoliation to overcome the low *R* (0.4–6.25 A·W^−1^) of PtSe_2_ [94]. The photogenerated holes trapped in the Se defects gated the platinum diselenide (PtSe_2_) channel (Figure 2b) and prolonged the electron lifetime, leading to a high *R* of 5 × 10^4^ A·W^−1^. This value of the photodetector with few-layer PtSe_2_ flakes exhibited four orders of magnitude higher than that in previous studies. Furthermore, NPC and positive photoconductance were observed depending on the V_G_ under light irradiation. As shown at point B in Figure 2c, a positive photocurrent was obtained by the electrons, the majority carrier. The opposite mechanism is applied at point A by holes. The photogating effect can also be utilized on a photodiode. The strong *R* and rapid response time in 2D van der Waals (vdW) heterostructures are incompatible because of their relatively weak optical absorption characteristic and weak photogenerated carrier dissociation force [95,96]. In 2019, Wang et al. addressed this problem by fabricating a CH_3_NH_3_PbI_3_ (MAPbI_3_)/black phosphorus (BP)/MoS_2_ photodiode (Figure 2d) [88]. The rapid response of the BP/MoS_2_ photodiode and the high *R* of the perovskite complemented each other, reaching *R* of 11 A·W^−1^ at a reverse bias of −2 V under 457 nm (Figure 2e). The device detected a broadband of light ranging from visible to NIR light, even under zero bias conditions, owing to the relatively small exciton binding energy of MAPbI_3_ (Figure 2f). Black phosphorus has attracted attention because of its high carrier mobility, low dark current, low noise photodetection due to direct bandgap energy ranging from 0.3 eV to 1.2 eV, compatible with various substrates, and strong photon absorption than monolayer 2D materials [97,98,99,100]. BP has a unique polarization characteristic that distinguishes it from other 2D materials because of its strong and intrinsic in-plane anisotropic properties [101,102]. In 2016, Guo et al. proposed a BP-based photodetector capable of operating over a wide wavelength range (532 nm–3.39 μm) at low picowatts power (Figure 2g) [103]. First, they confirmed the relation between light polarization and carrier collection directions according to I_Ph_. When both the polarization and carrier collection directions were along the *x*-(armchair) direction, the I_ph_ value was a factor of three larger than when both were along the *y*-(zigzag) direction. The polarization of scattered light can convey various information, such as the surface roughness, morphology, and orientation of objects, even under hazy/foggy conditions [104,105]. The photogating mechanism working in the device was elucidated. The V_G_ at the I_Ph_ peak, as shown in Figure 2h, was close to the V_Th_ rather than the minimum conductance voltage. When the V_G_ around V_Th_ was applied but the device was still in on-state, I_Ph_ was maximized as the carrier transition time became shortened. However, when the device was in the off-state as V_G_ larger than V_Th_ was applied, I_Ph_ was decreased since both the hole and electron trap states were able to capture the corresponding carriers. The device showed a high *R* of up to 82 A·W^−1^ even at room temperature under 3.39 μm of light irradiation.

### 2.3. CNT-Based Photodetectors

**Figure 3:**
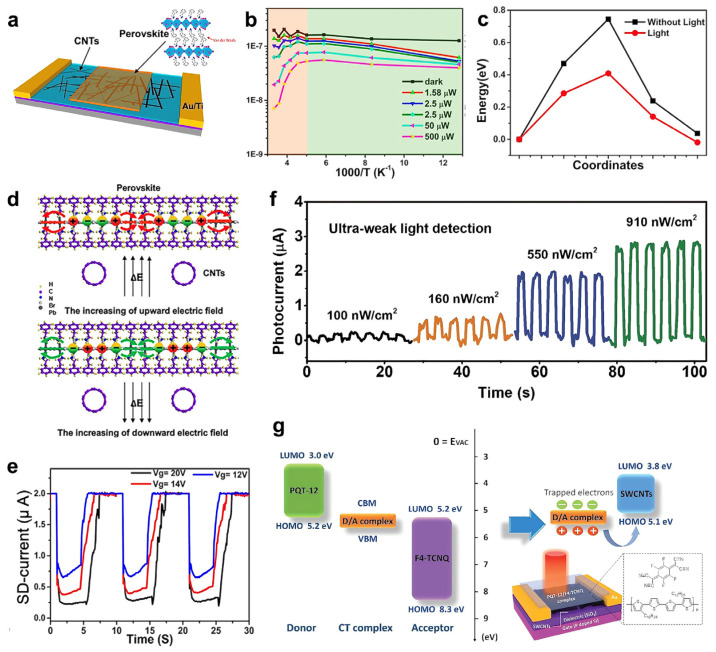
CNT-based photodetector. (**a**) Scheme of a perovskite/CNTs heterojunction transistor. (**b**) I_D_ as a function of temperature under various intensities of light at a fixed V_G_ of −30 V. (**c**) Ion migration barrier of perovskite with and without light illumination. (**d**) Schematic diagram of in-plane charge migration in lateral direction of perovskite under an electric field. (**e**) I_D_ as a function of time with different V_G_ showing application as photo-memory. (**f**) I_Ph_ of the device irradiating ultra-weak intensities of 100, 160, 550, and 910 nW·cm^−2^ light, respectively. (**g**) Band diagram of the donor, acceptor, D/A complex, and SWCNTs film, and the scheme of charge transfer under infrared light. Inset: schematic diagram of the device. (**a**–**e**) Reproduced with permission from [106]. Copyright Springer Nature, 2019. (**f**,**g**) Reproduced with permission from [109]. Copyright Wiley, 2021.

A two-dimensional (2D) halide perovskite forming quantum-well structure is drawing attention for its low-cost efficiency, unique optical property, and moisture stability [4]. However, the ion migration characteristic in halide perovskite induces a long carrier lifetime. Li et al. showed a negative photogating effect of the heterostructure FET by utilizing the structure of CNT and its feasibility as photo-memory (Figure 3a) [106]. The I_D_ of the device was reduced with the increase of 470 nm light intensity (dark to 500 μW) illumination, showing the negative photogating effect (Figure 3b). As the photo-excited electrons migrated into the conduction band, halogen vacancies in the perovskite also migrated through the pathway. It led to a decrease in the diffusion barrier and an increase in the dielectric constant (Figure 3c). The cylindrical structure of CNTs caused potential redistributions, and consequently resulted in V_G_ screening on CNTs by (PEA)_2_PbI_4_ (Figure 3d). The migrated ions under light irradiation were captured in a quasi-steady state when back in a dark condition, suggesting a possibility as an optical memory device (Figure 3e). Despite carbon nanotubes (CNTs) being highly light absorbent materials with excellent carrier mobility, binding energy resulting from the unique 1D structure makes them suffer from low operating speed [107,108]. In 2021, Yang et al. implemented IR phototransistors with a PQT-12 (poly (3,3‴-dialkylquaterthiophene)/F4-TCNQ (2,3,5,6-tetrafluoro-7,7,8,8-tetracyanoquinodimethane) donor/acceptor (D/A) as a photoactive layer and single-walled carbon nanotubes (SWCNTs) as a channel layer [109]. The IR phototransistor has shown its feasibility in various fields, such as blackbody detectors, flexible phototransistors, and synaptic devices [110]. The electronic transition energy (0.4 eV) of the D/A complex is due to charge transfer excitation attributed to the broadband photodetection extending from the visible region to the NIR region (400–2600 nm). An *R* of 2.75 × 10^6^ A·W^−1^ and detectivity (D*) of 3.12 × 10^14^ Jones under 2000 nm were obtained with good stability and repeatability. The device responded even when ultra-weak 100 nW·cm^−2^ light was illuminated (Figure 3f). Under light irradiation, photogenerated holes were injected from the D/A complex to the SWCNTs layer, while photogenerated electrons were trapped at the trap sites present in the D/A complex, defects, or complex/SWCNTs interfaces (Figure 3g).

### 2.4. Inorganic Compound-Based Photodetectors

**Figure 4:**
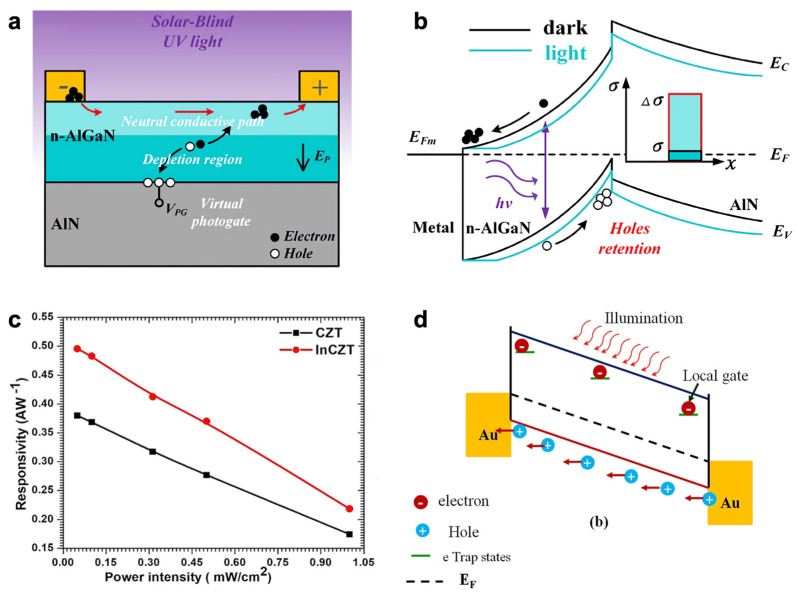
Inorganic compound-based photodetector. (**a**) Schematic diagram of an Al_0.5_Ga_0.5_N∕AlN solar-blind UV photodetector and its neutral conductive path under DUV irradiation. (**b**) Schematic diagram of the energy band under dark and illumination conditions. (**c**) Measured and fitted photocurrent of a CZT- and InCZT-based device under different intensities of light irradiation. (**d**) Schematic of the band diagram of the device under light illumination. (**a**,**b**) Reproduced with permission from [113]. Copyright Chinese Laser Press, 2022. (**c**,**d**) Reproduced with permission from [116]. Copyright American Chemical Society, 2019.

The wide bandgap (>3.4 eV of gallium nitride, GaN) of semiconductor materials, including gallium(III) oxide (Ga_2_O_3_), Mg_x_Zn_1−x_O, and III-nitride compounds (Al_x_Ga_1−x_N/AlN and BN), are emerging as the next generation materials for solar-blind ultraviolet (SBUV) photodetectors [111,112]. In 2022, Lu et al. proposed a solar-blind ultraviolet (UV) detector with high photodetection performance and a simple structure and process (Figure 4a) [113]. Spontaneous and piezoelectric polarization of n-Al_0.5_Ga_0.5_N generated a perpendicular electric field (E_P_), which contributes to an increase in I_Ph_ because of the photogating effect. Under light irradiation, E_P_ dissociated the photoinduced excitons in the depletion channel region into electrons and holes, respectively. The negatively charged interface accumulated holes, leading to less band bending and more electron injection, thus enhancing the photoconductivity (Figure 4b). The ultrafast rise time of 537.5 ps and *R* of 10^5^ A·W^−1^ at 20 V bias were obtained, which took a fast response speed and high *R*-value. The I_On_/I_Off_ ratio of 10^4^ at a very weak intensity of 0.7 nW·cm^2^ further showed its potential for use as a flame detector. Cadmium zinc telluride (CdZnTe or CZT) is an emerging material as a radiation detector owing to its wide band gap of ~1.68 eV, large photon absorption area, high resistivity of 10^10^ Ω·cm, and environmental stability [114,115]. However, its applications are limited due to impurities and inherent defects, which lead to the low hole mobility. Shkir et al. accomplished a balanced carrier concentration by inserting indium into the CZT crystal as an additional donor impurity [116]. The photogating effect caused by these additional defects led to a low I_On_/I_Off_ ratio and D*, while there was an increase in *R* and EQE compared to those of intrinsic CZT (Figure 4c). Under light irradiation, the photogenerated electrons trapped at the trap states acted as an additional gate voltage bias, reducing the resistivity of devices (Figure 4d). On the other hand, the photogenerated holes can transit many cycles before the recombination, leading to a high EQE. They showed a new area of research based on CZT crystals for future visible photodetector devices utilizing the photogating effect, along with improving the I_On_/I_Off_ ratio or D*.

### 2.5. Organic-Based Photodetectors

**Figure 5:**
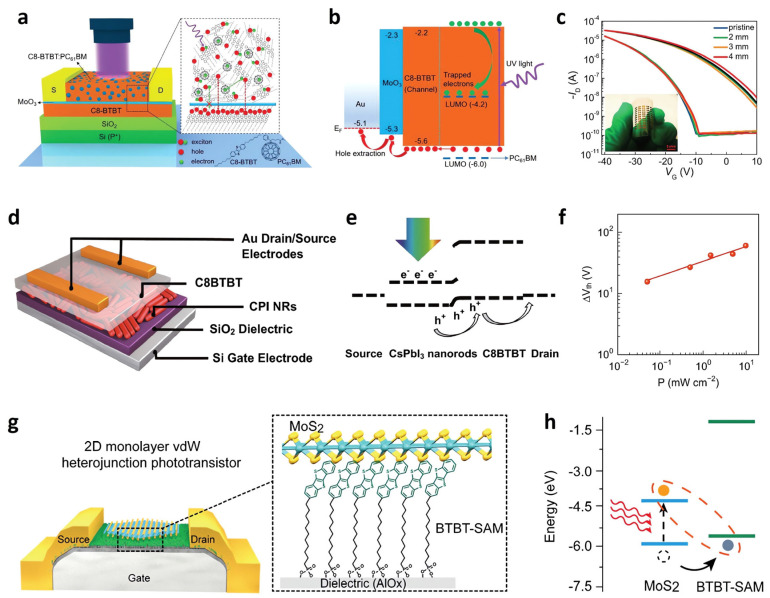
Organic-based photodetector. (**a**) Schematic illustration of the HL-OPT. (**b**) Energy band diagram of the HL-OPT device under UV illumination. (**c**) Transfer characteristics of the flexible HL-OPT with various bending radii with and without UV light. (**d**) Schematic illustration of the C8BTBT/CsPbI_3_ nanorod-based phototransistor. (**e**) Schematic diagram of a photogenerated carrier flow in the hybrid phototransistor under light irradiation. (**f**) V_Th_ shift of the hybrid phototransistor as a function of light intensity. (**g**) Scheme of the 1L-MoS_2_/BTBT-SAM heterostructure device. (**h**) Energy band diagram of the 1L-MoS_2_ and BTBT-SAM under light irradiation. (**a**–**c**) Reproduced with permission from [16]. Copyright Wiley, 2019. (**d**–**f**) Reproduced with permission from [120]. Copyright Springer, 2018. (**g**,**h**) Reproduced with permission from [126]. Copyright Wiley, 2021.

Organic semiconductors have great potential in future optoelectronic and flexible devices with easy bandgap tunability, fabrication simplicity, cost efficiency, large-area processability, and resolvability [117,118,119]. 2,7-dioctyl[1]benzothieno[3,2-b][1]benzothiophene (C8-BTBT) is used widely because of its stability in air and its carrier mobility [120,121]. The newly proposed HL-OPT structure consisted of a channel layer of C8-BTBT, a photoactive D/A layer of C8-BTBT:PC_61_BM hybrid material, and an interlayer of molybdenum trioxide (MoO_3_) (Figure 5a). The charge-selective interlayer assists hole injection into the channel and prevents electrons from recombining with the holes. The interlayer physically separates the channel layer and photoactive layer, improving photodetection performance. The photoinduced electrons are trapped at the PC_61_BM, enhancing the hole concentration of the channel by the photogating effect (Figure 5b). As a result, *R* = 8.6 × 10^3^ A W^−1^ and D* = 3.4 × 10^14^ Jones were obtained, even under weak UV irradiation (intensity of 32 μW·cm^−2^). Furthermore, the device detected UV successfully, even on a bendable polyethylene terephthalate (PET) substrate (Figure 5c). All-inorganic cesium lead halide (CsPbX_3_) (X = I, Br, and Cl) perovskites have a moderate bandgap, weakly bound excitons, high absorption coefficient, long carrier lifetime, and low-cost fabrication [122,123]. On the other hand, their unstable phase transition has limited their applications [124,125]. Phase stability could be achieved by reducing its dimension to the nanoscale. In 2018, Chen et al. fabricated a heterostructure photodetector with C8-BTBT and a dip-coated cesium lead iodide (CsPbI_3_) nanorod (NR) thin film (Figure 5d) [120]. The energy level difference between C8-BTBT and CsPbI_3_ NR, forming a type- II heterojunction, enabled efficient photogenerated hole transport and protected photogenerated charge from recombination. When white LED was irradiated, photocarriers were generated in the CsPbI_3_ and dissociated near the CsPbI_3_/C8-BTBT interface. The photogenerated holes transferred to the channel layer, while the electrons were trapped at the CsPbI_3_, acting as an extra negative gate bias (Figure 5e). V_th_ increased as a function of the light intensity, indicating the photogating effect, as well (Figure 5f). An *R* up to 4.3 × 10^3^ A·W^−1^ was obtained. In 2021, Zhao et al. devised a unique strategy using a [12-(benzo[b]benzo[4,5]thieno[2,3-d]thiophen-2-yl)dodecyl)]phosphonic acid self-assembled monolayer (BTBT-SAM) for the all-2D hybrid organic–inorganic vdW heterojunction phototransistors (Figure 5g) [126]. They transferred the monolayer MoS_2_ (1L-MoS_2_) crystal onto a uniform surface of the organic thin films. The photogenerated holes are transferred to the BTBT-SAM layer outside the MoS_2_ crystal region, while the photogenerated electrons are left inside the crystal (Figure 5h). The device showed an *R* of 475 A·W^−1^, ascribed to the photogating effect.

### 2.6. Oxide-Based Photodetectors

**Figure 6:**
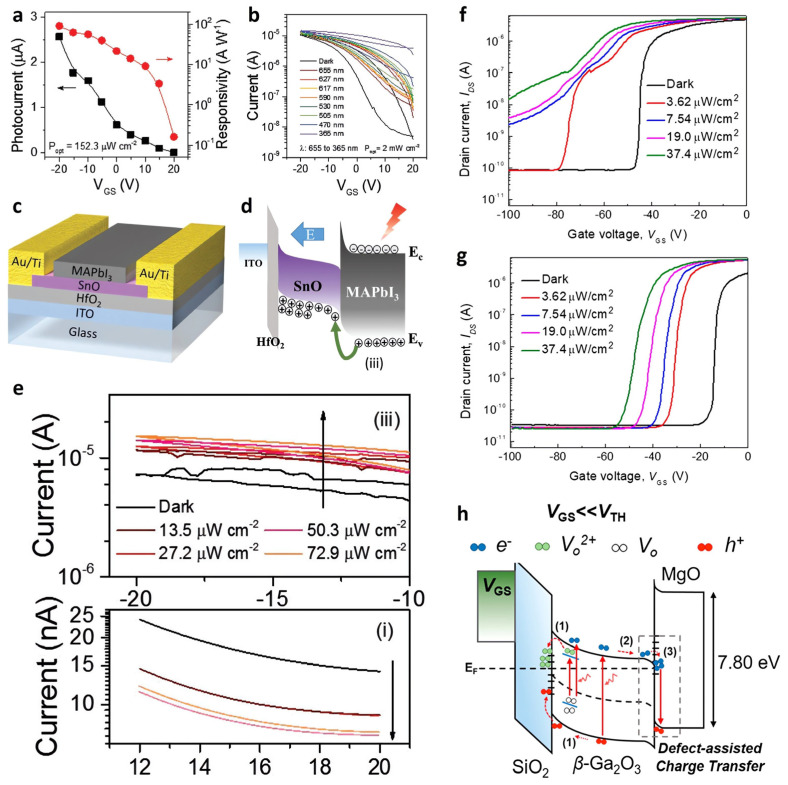
Oxide-based photodetector. (**a**) Plots of I_Ph_ and *R* as a function of V_G_ at the light intensity. (**b**) Transfer curves of the phototransistor with different wavelengths of light illumination. (**c**) Schematic diagram of the SnO phototransistor. (**d**) Schematic diagram of the charge transport mechanism in the SnO/perovskite interface under light irradiation, corresponding to the upper panel of Figure 5e. (**e**) Magnified transfer characteristics of the perovskite/SnO phototransistor at specific V_G_. Transfer characteristics of the (**f**) β-Ga_2_O_3_ and (**g**) β-Ga_2_O_3_/MgO heterostructure-based phototransistors. A light-induced hump appeared in the β-Ga_2_O_3_ phototransistor when the V_G_ was lower than V_Th_. (**h**) Schematic energy band and carrier transport at the β-Ga_2_O_3_/MgO device when the V_G_ was less than V_Th_. (**a**–**e**) Reproduced with permission from [127]. Copyright Wiley, 2019. (**f**–**h**) Reproduced with permission from [130]. Copyright American Chemical Society, 2021.

In 2019, Guan et al. introduced tin monoxide (SnO) as a new *p*-type oxide-based phototransistor [127]. Although the SnO-based device itself exhibited a good photodetection performance with *R =* 1.83 × 10^3^ A·W^−1^ (Figure 6a) and a broadband response from the UV to visible range (365–655 nm) (Figure 6b), its moderate light absorption characteristic drew attention to other methods. Therefore, a photodetector with better performance was realized by coating hybrid perovskite MAPbI_3_ as a photoactive layer for a smooth charge transfer from perovskite to *p*-type SnO (Figure 6c). MAPbI_3_ only covered the channel layer and did not contact the electrodes. When exposed to white light, the on-state current increased, and the off-state current decreased compared to the dark state. When V_G_ > 0 V (panel (i) in Figure 6e), the I_Off_ was decreased because of charge recombination as the electrons generated in MAPbI_3_ were transported to the SnO, and because of the photogating effect, as photogenerated holes remained in the perovskite layer. Because the opposite carrier movement was observed when V_G_ < 0 (panel (iii) in Figure 6e), the photogenerated holes migrated to the SnO, and the photogenerated electrons remained in the MAPbI_3_. The additional negative gate bias was ascribed to the photogating effect and the increased hole injection, leading to an increase of the I_On_ (Figure 6d). Therefore, the I_On_/I_Off_ ratio increased from 519 to 2674, and μ increased from 3.46 to 5.53 cm^2^·V^−1^·s^−1^. Wide-band-gap semiconductor-based optoelectronics respond in the deep-ultraviolet (DUV) range, bringing their solar-blind properties to industrial, biological, environmental, and military applications [128,129]. In 2021, Ahn et al. chose β-Ga_2_O_3_ as the photo-absorption and channel layer, which provides a detection wavelength at 230–280 nm [130]. By adding Al_2_O_3_ encapsulated MgO layer on top of the device, the device showed no hump in transfer curves unlike the device without MgO layer (Figure 6f,g). A high *R* and D* of 2.3 × 10^7^ A·W^−1^ and 1.7 × 10^15^ Jones, respectively, were obtained with the β-Ga_2_O_3_/MgO heterostructure-based phototransistor. This was possible because of the defect-assisted charge transfer mechanism. When V_G_ was smaller than V_Th_, photo-excited hole carriers and oxygen ion vacancies were accumulated at the deep oxygen defect trap sites in the SiO_2_/β-Ga_2_O_3_ interface, shifting the V_Th_ to a negative gate bias (process 1 in Figure 6h). At the same time, the photogenerated electrons were accumulated at the β-Ga_2_O_3_/MgO junction, suppressing the photoconductive effect and only allowing the photogating effect to appear (process 2 in Figure 6h).

## 3. Application

The photogating effect has been used mainly to improve photodetection performance. On the other hand, recent studies have been reported in which the photogating effect further plays a role as a key mechanism of application. In particular, it began to be applied in neuromorphic devices [131,132,133,134] and optoelectronic memory [135,136,137], focusing on the photogating effect and the PPC behavior.

**Figure 7:**
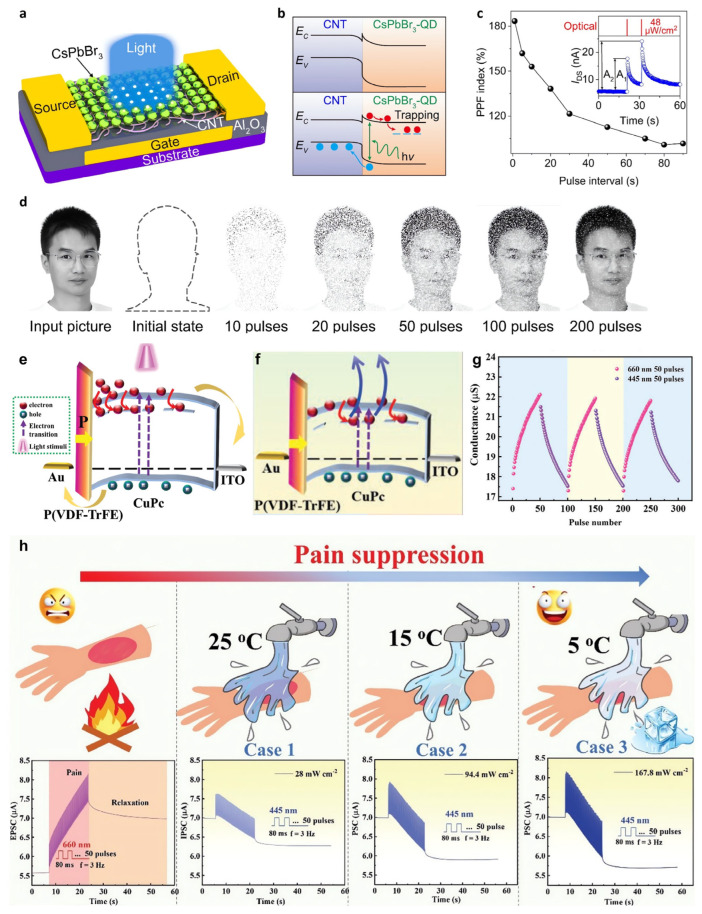
Image sensor and neuromorphic device utilizing the photogating effect. (**a**) Schematic diagram of the phototransistor with a CNT/CsPbBr_3_-QD channel. (**b**) Energy band diagram at the dark (upper panel) and illumination states (lower panel). (**c**) PPF index decreased continuously as a reverse function of the pulse interval. Inset: PPF results from two consecutively applied light pulses. (**d**) Human face learning simulation results with the sensor array in the initial state and after training processes by applying 0, 10, 20, 50, 100, and 200 light pulses (1 μW·cm^2^). (**e**) Band diagram scheme of the device when P(VDF-TrFE) is downward polarized. (**f**) Band diagram scheme of the device when 445 nm of light is irradiated. (**g**) Three successive cycles of potentiation and depression curves by irradiating 660 and 445 nm light pulses, respectively. (**h**) Current changes according to the 28 μW·cm^−2^, 94.4 μW·cm^−2^, and 167.8 μW·cm^−2^ of light pulse intensities: simulating pain formation and suppression processes of nociceptors according to 25, 15, and 5 °C of water temperature. (**a**–**d**) Reproduced with permission from [138]. Copyright Springer Nature, 2021. (**e**–**h**) Reproduced with permission from [139]. Copyright Wiley, 2022.

Neuromorphic device studies to mimic the human brain operating at ultra-low power consumption and ultra-high efficiency have been explored to solve the bottleneck problem of the von Neumann architecture. In 2021, Zhu et al. exploited the photogating effect in an optoelectronic sensor for future potential artificial vision systems [138]. They implemented a flexible CNTs/CsPbBr_3_-quantum dots (QDs) heterostructure-based optoelectronic sensor array with 1024 pixels (Figure 7a). The photogenerated holes were dissociated with electrons in CsPbBr_3_-QDs and dispersed to the CNTs, while the remaining photogenerated electrons were trapped in the QD layer. When the light pulse was applied, the trapped electrons induced more hole carriers through capacitive coupling, increasing the current flow in the channel through the photogating effect (Figure 7b). The device exhibited *R* = 5.1 × 10^7^ A·W^−1^ and a D* = 2 × 10^16^ Jones. In addition, the long electron decay time induced PPC behavior in the device. When a second optical pulse was applied, more electrons were trapped because of the higher electric field, resulting in a higher current (Figure 7c). Furthermore, 0, 10, 20, 50, 100, and 200 pulses under 1 μW·cm^2^ of weak light were applied for training a sensor array to mimic the recognition process of a human face (Figure 7d). As the number of pulses increased, learning of the facial features was reinforced. The nociceptor is a sensory neuron that perceives pain according to external stimuli. Based on the bidirectional responses, it receives and transmits signals to the spinal cord and brain. After perceived pain in the nociceptor, the relaxation process proceeded by decreasing the signal. In 2022, Ji et al. fabricated a bidirectional synaptic device with organic materials, which are copper-phthalocyanine (CuPc) and poly(vinylidene fluoridetrifluoroethylene) (P(VDF-TrFE)) [139]. Au electrodes, a P(VDF-TrFE)/CuPc heterostructure semiconductor, and an ITO electrode served as the presynaptic membranes, synaptic cleft, and postsynaptic membrane, respectively. Various synaptic performances, such as excitatory or inhibitory postsynaptic current (EPSC/IPSC), paired-pulse facilitation (PPF), spiking-rate-dependent plasticity (SRDP), short- or long-term potentiation (STP/LTP), spike-number-dependent-plasticity (SNDP), and spike-width-dependent-plasticity (SWDP), were fully modulated with light. P(VDF-TrFE) is a ferroelectric material that is polarized by an external electric field. Therefore, the PPC behavior was dependent on the presence of P(VDF-TrFE) and its polarization direction when irradiated with light of 660 nm, the wavelength spectrum to which CuPc responds. The EPSC decayed slowly when the polarization direction of P(VDF-TrFE) was in a downward state rather than in an upward state or no direction. This was attributed to the strong photogating effect in which the energy barrier in the P(VDF-TrFE)/CuPc interface interfered with the dissociation of nonequilibrium holes into the electrode (Figure 7e). Interestingly, successive increases and decreases in I_Ph_, leading to EPSC and IPSC were controlled by applying 660 nm and 445 nm of light, respectively (Figure 7g). This bidirectional photoresponsive characteristic enabled the device to mimic the action and relaxation processes of the nociceptor in an entirely optical manner (Figure 7h). The energy of 445 nm light attenuated the photogating effect by reducing the number of trapped electrons, resulting in the NPC effect (Figure 7f).

**Figure 8:**
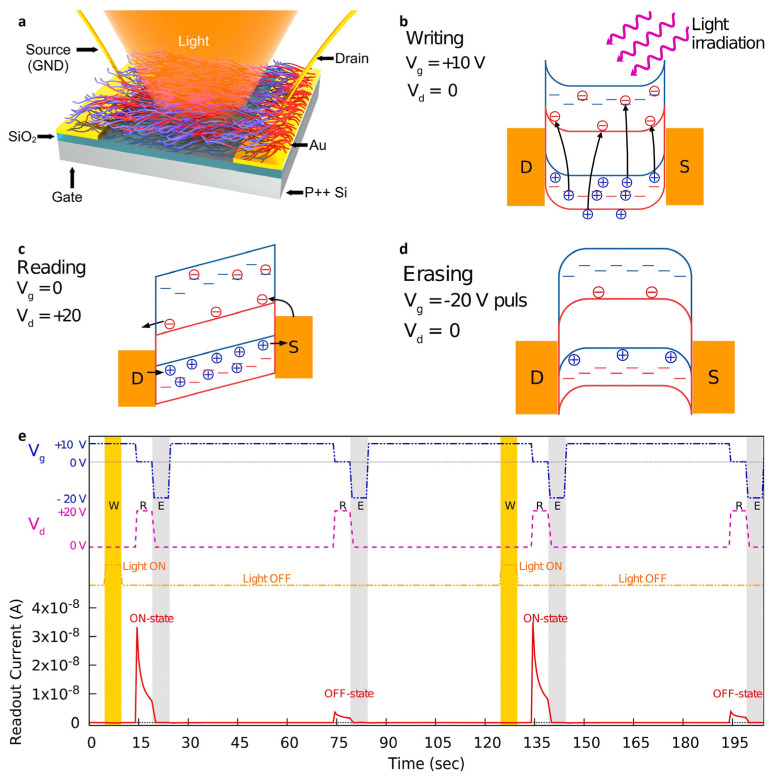
Optoelectronic memory device using the photogating effect. (**a**) Schematic diagram of the P(BDBT-co-N2200)-based phototransistor structure. Schematic diagrams of the energy band and charge transport of the optoelectronic memory in (**b**) writing, (**c**) reading, and (**d**) erasing processes; the red box and dotted line represent the energy band and trap states of N2200, respectively; the blue box and dotted line represent the energy band and trap states of PBDB-T, respectively. (**e**) Sequence process of writing (W), reading (R), and erasing (E) operations for P(BDBT-co-N2200)-based optoelectronic memory. (**a**–**e**) Reproduced with permission from [143]. Copyright Elsevier, 2022.

Conventional memory is no longer sufficient to meet the modern needs of ultrahigh-density data storage, such as multilevel storage, data encryption, image capturing, information recording, logic data processing, and wearable sensors [140,141,142]. Therefore, new memory devices that transmit information quickly with low energy consumption are in demand. In 2022, Kim et al. reported an ambipolar photo-memory-transistor using a π-conjugated *p*-type Poly[(2,6-(4,8-bis(5-(2-ethylhexyl)thiophen-2-yl)-benzo [1,2-b:4,5-b′]dithiophene))-alt-(5,5-(1′,3′-di-2-thienyl-5′,7′-bis(2-ethylhexyl)benzo [1′,2′-c:4′,5′-c′]dithiophene-4,8-dione)] (PBDB-T) and *n*-type N2200 macromolecular copolymer (P(BDBT-co-N2200)) (Figure 8a) [143]. The transistor exhibited high hysteresis because of the interfacial trap states. These intrinsically localized quantum states are according to the amorphous phase of the D/A heterojunction. On the other hand, hysteresis was reduced and I_Ph_ was increased when irradiated with light because of the photogating effect by the charges trapped at the trap states. The trapped charge carriers determined the writing (W) (Figure 8b), reading (R) (Figure 8c), and erasing (E) (Figure 8d) states of the optoelectronic memory device by adjusting the V_G_ and light irradiation conditions, respectively (Figure 8e). The device showed reliable storage performance with a constant readout current owing to the relatively strongly trapped carriers.

## 4. Summary and Outlook

This review provided a comprehensive insight on photogating-based photodetectors. Photogating reviews focused mainly on 2D material-based photodetectors were expanded in this paper to various active materials, including perovskite, organics, compounds, polymers, and oxides, as well as 2D materials. The strategies to improve the photodetection performance, the structure, material, and mechanism were organized and classified by channel material. Most of the studies utilized *p*-type semiconductors as a channel, and implemented heterostructures to increase the defects or vacancies and modulate the energy band. Some studies utilized the polarization characteristic of materials for potential redistribution. The features are summarized in Table 2 [5].

Furthermore, efforts to implement the photogating effect with emerging applications in neuromorphic devices and optoelectronic memories were revisited. We expect that the photogating effect will be used more actively without material limitation, helping to realize applications of various fields, as well as memory and neuromorphic devices. Nonetheless, still there are difficulties to be overcome, as follows:

(i) Operational stability in photogating effect-based devices is an issue that must be addressed. Since the photogating effect forms an additional gate bias by the trapped carrier, bias stress instability should exist due to the trapped charges. To allow these devices to be used as practical applications, an effective strategy that exhibits the photogating effect with operational stability is absolutely necessary.
(ii) There is a need to explore more various active materials, as previous research efforts tended to focus on 2D materials for implementing the photogating effect and its application. As another material aspect, efforts on n-type materials-based photogating effect devices are lacking. Most of the photogating effect-based devices are based on *p*-type materials, such as WSe_2_, MoS_2_, and C8-BTBT, and, thus, the use of the counterpart *n*-type materials is required.
(iii) To develop high-performance photogating effect-based photodetection, it must be designed in consideration of transistor characteristics. Basically, light exposure is distinguished by a V_Th_ shift in this kind of the photogating effect-based devices. For this reason, transistor parameters, such as dielectric and semiconductor layer thickness, surface interface characteristics, source drain, and gate electrode work function, must be simultaneously considered to effectively change V_Th_ by the photogating effect.
(iv) Efforts on reproducibility and uniformity should be made to extend these photogating effect-based devices to more diverse applications. Large-scale and robust integration, which is required for advanced applications, can be obtained by the assurance of the technology reproducibility and uniformity.

Although the above-described challenges are required to be overcome, the potential of the photogating effect is clear; detection of sub-bandgap rays is available and high sensitivity photodetection can be obtained by the shift of V_Th_. Based on the revisited advances in recent photogating effect-based devices, this review suggests the possibility that the photogating effect can be used as another alternative mechanism for the development of next-generation optoelectronic devices.

## Figures and Tables

**Table 1:** Summary of several physical quantities in photodetector.

Parameter	Explanation	Formula	Unit
Photocurrent (*I_ph_*)	A current when light is irradiated; a clear signal detection is determined by a high photocurrent value.	NA	A
Responsivity (*R*)	A sensitivity of the device according to the intensity of light illumination.	$\frac{I_{ph}-I_{d}}{P_{opt}A}$	A·W^−1^
Noise equivalent power (*NEP)*	The weakest light signals a device can detect; the smaller the NEP, the better the performance of the detector.	$\frac{g_{n}}{R}$	W·Hz^−1/2^
Specific detectivity (D*)	Inversion of the NEP, which indicates the relative noise level present in the device.	$\frac{\sqrt{A\Delta f}}{NEP}$	Jones
External quantum efficiency (*EQE*)	The efficiency of the converted charge carrier flux to the incident photon flux.	$\frac{Rh\nu }{e}$	Unitless
Response time (τ)	The time that it takes for the photodetector to change the output as the input light intensity changes.	$\frac{1}{2\pi f_{T}}$	s

**Table 2:** Various active channel materials for the photodetection performances.

Channel Material		Type	Wavelength	*R*_max_ [A·W^−1^]	D* [Jones]	Structure	Mechanism/ Feature	Application	Ref.
Graphene		*p*	532–980 nm	0.26 (635 nm)	NA	Hetero Asymmetric	Conductivity modulation	NA	[80]
		Ambipolar	4.6 μm	33.8 (81.6 μW·cm^−2^)	NA	Bare	Substrate change	NA	[83]
		*p*	975 nm	20 (50 nW)	NA	Hetero	Conductivity modulation	NA	[84]
TMD	WSe_2_	*p*	white light LED	3.6 × 10^3^ (1.1 nW)	NA	Bare	Defect engineering	NA	[91]
	PtSe_2_	Ambipolar	405 nm	5 × 10^4^ (0.13 μW·cm^−2^)	3 × 10^7^	Bare	Vacancy engineering	NA	[94]
	MoS_2_	*p*	457–808 nm	11 (0.02 μW·cm^−2^)	1.3 × 10^12^	Hetero	Energy band/ Defect engineering	NA	[88]
BP		*p*	532 nm– 3.39 μm	82 (3.39 μm)	NA	Bare	Polarization/ Energy band engineering	NA	[103]
CNT		*p*	400–2600 nm	2.75 × 10^6^ (2000 nm)	3.12 × 10^14^ (2000 nm)	D/A hetero	Defect engineering	Blackbody radiation measurement Flexible Neuromorphic device	[109]
		*p*	470 nm	N/A	N/A	Hetero	Potential redistribution	Optoelectronic memory	[106]
Inorganic compound	n-Al_0.5_Ga_0.5_N	*n*	213–280 nm	1.6 × 10^5^ (240 nm)	1.52 × 10^18^	Bare	Polarization/ Potential redistribution	NA	[113]
	InCZT	*p*	632.8 nm	0.50 (0.049 mW·cm^−2^)	1.80 × 10^11^	Bare	Defect engineering	NA	[116]
Organic	C8-BTBT	*p*	352–700 nm	8.6 × 10^3^ (365 nm)	3.4 × 10^14^	D/A hetero	Interlayer insertion	Flexible	[16]
	C8-BTBT	*p*	White light	4.3 × 10^3^	NA	Hetero	Energy band engineering	NA	[120]
	BTBT-SAM	*p*	406 nm	475	NA	Hetero	Material treatment	NA	[126]
Oxide	SnO	*p*	365–655 nm	1.83 × 10^3^ (655 nm)	2.11 × 10^13^	Hetero	Energy band engineering	NA	[127]
	β-Ga_2_O_3_	*n*	260 nm	2.3 × 10^7^ (0.51 μW·cm^−2^)	1.7 × 10^15^	Hetero	Defect engineering	NA	[130]
CNT		*p*	405 nm and 516 nm	5.1 × 10^7^ (0.01 μW·cm^−2^)	2 × 10^16^	Hetero	Potential redistribution	Optoelectronic sensor Flexible	[138]
Inorganic compound	CuPc	*p*	445 nm and 660 nm	NA	NA	Hetero	Polarization/ Potential redistribution	Neuromorphic device	[139]
Copolymer	P(BDBT-co-N2200)	Ambipolar	505 nm, 627 nm, and 735 nm	NA	NA	D/A hetero	Defect engineering	Optoelectronic memory	[143]

## Data Availability

Not applicable.
